# The genome sequence of the fork-jawed nomad bee, *Nomada ruficornis *(Linnaeus, 1758)

**DOI:** 10.12688/wellcomeopenres.19928.1

**Published:** 2023-09-06

**Authors:** Steven Falk, Joseph Monks, University of Oxford and Wytham Woods Genome Acquisition Lab, Darwin Tree of Life Barcoding collective, Wellcome Sanger Institute Tree of Life programme, Wellcome Sanger Institute Scientific Operations: DNA Pipelines collective, Tree of Life Core Informatics collective, Darwin Tree of Life Consortium

**Affiliations:** 1 Independent researcher, Kenilworth, England, UK; 2 Natural History Museum, London, England, UK

**Keywords:** Nomada ruficornis, fork-jawed nomad bee, genome sequence, chromosomal, Hymenoptera

## Abstract

We present a genome assembly from an individual male *Nomada ruficornis* (the fork-jawed nomad bee; Arthropoda; Insecta; Hymenoptera; Apidae). The genome sequence is 273.0 megabases in span. Most of the assembly is scaffolded into 16 chromosomal pseudomolecules. The mitochondrial genome has also been assembled and is 20.66 kilobases in length.

## Species taxonomy

Eukaryota; Metazoa; Eumetazoa; Bilateria; Protostomia; Ecdysozoa; Panarthropoda; Arthropoda; Mandibulata; Pancrustacea; Hexapoda; Insecta; Dicondylia; Pterygota; Neoptera; Endopterygota; Hymenoptera; Apocrita; Aculeata; Apoidea; Anthophila; Apidae; Nomadinae; Nomadini; Nomada (Linnaeus, 1758) (NCBI:txid601849).

## Background

Bees from the *Nomada* genus are commonly called Nomad Bees since they frequently roam close to the ground in search of their host’s nests. All *Nomada* species practise kleptoparasitism, depositing their eggs in other bees’ nests and utilizing the pollen gathered by those host bees. The host species are often from the *Andrena* genus.

*Nomada ruficornis* Linnaeus is a medium-sized kleptoparasitic bee (females 8–11mm, males 7–11 mm). The species can be distinguished from all other UK *Nomada species*, excluding *N. fabricana* (Linnaeus), due to the bidentate mandibles, which has given it the common name of ‘fork-jawed nomad bee’.

*Nomada ruficornis* is univoltine, active from March to July ( Smit, 2018). The host is recorded as *Andrena haemorrhoa* (Fabricius), a ground nesting polylectic species. The host is common throughout the UK, nesting in a wide range of habitat types. In turn *N. ruficornis* is found throughout the UK but becomes more scarce further north. Outside of the UK, the distribution extends across the Palearctic to Japan ( Ascher & Pickering, 2016).

The genome of the fork-jawed nomad bee, *Nomada ruficornis*, was sequenced as part of the Darwin Tree of Life Project, a collaborative effort to sequence all named eukaryotic species in the Atlantic Archipelago of Britain and Ireland. Here we present a chromosomally complete genome sequence for *Nomada ruficornis*, based on one male specimen from Wytham Woods.

## Genome sequence report

**Figure 1.**
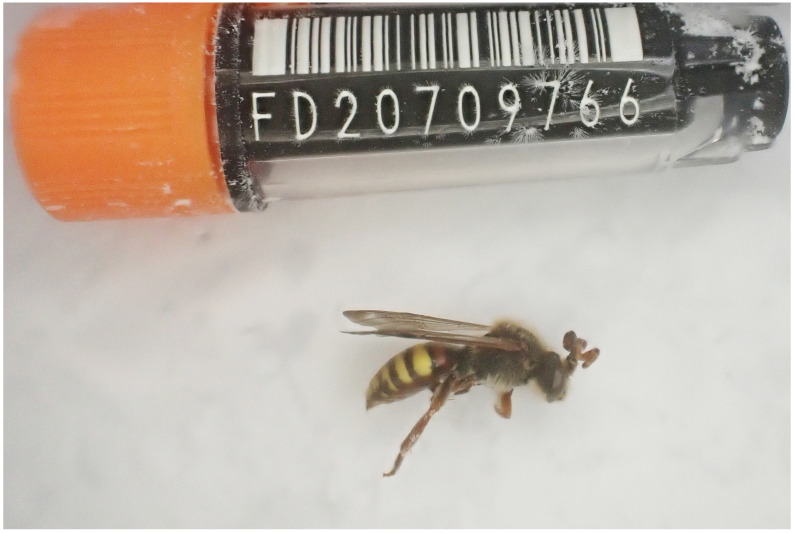
Photograph of the *Nomada ruficornis* (iyNomRufi1) specimen used for genome sequencing.

The genome was sequenced from one male *Nomada ruficornis* ( Figure 1) collected from Wytham Woods, Oxfordshire, UK (51.76, –1.34). A total of 92-fold coverage in Pacific Biosciences single-molecule HiFi long reads was generated. Primary assembly contigs were scaffolded with chromosome conformation Hi-C data. Manual assembly curation corrected 17 missing joins or mis-joins, reducing the scaffold number by 8.6% and increasing the scaffold N50 by 1.69%.

**Figure 2.**
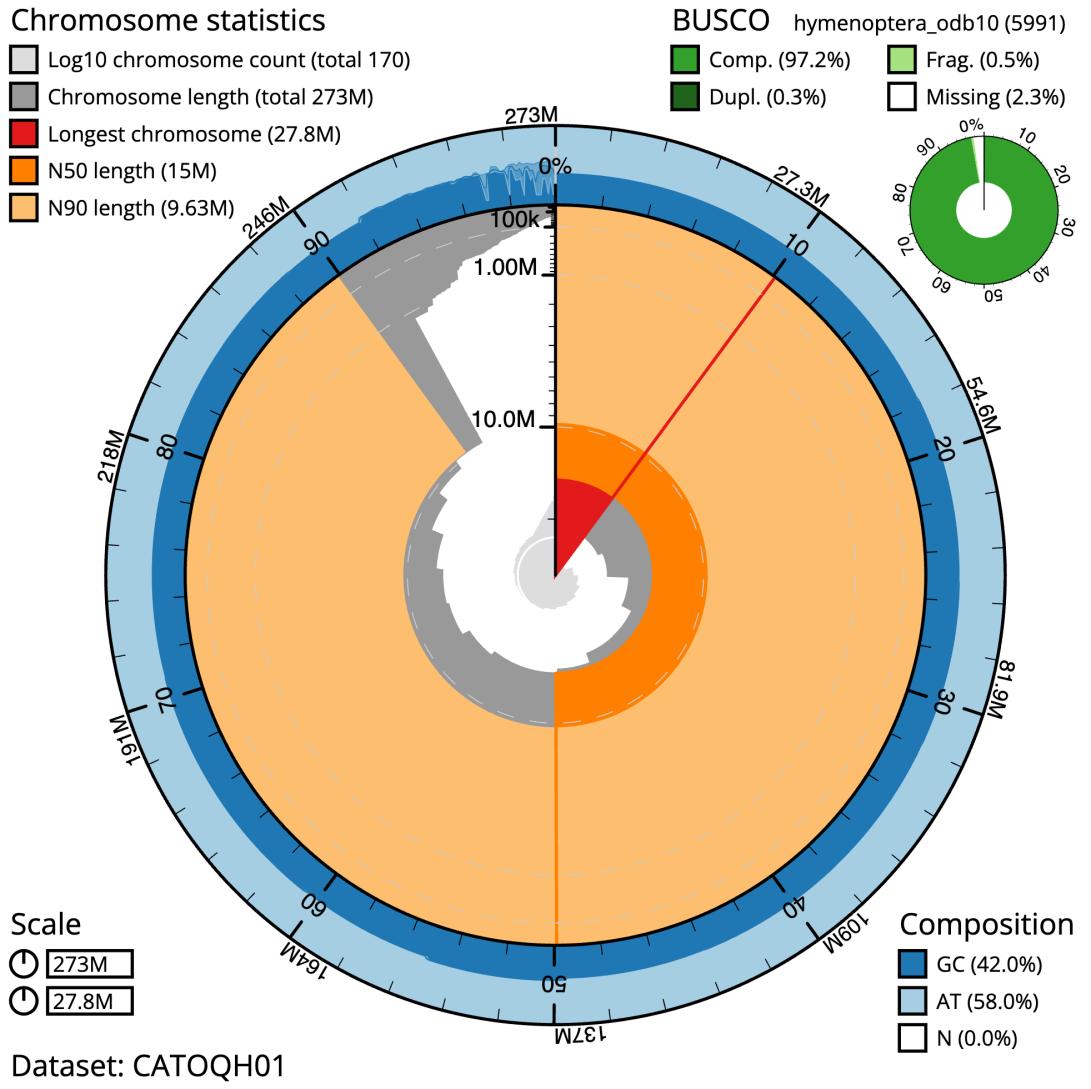
Genome assembly of *Nomada ruficornis*, iyNomRufi1.1: metrics. The BlobToolKit Snailplot shows N50 metrics and BUSCO gene completeness. The main plot is divided into 1,000 size-ordered bins around the circumference with each bin representing 0.1% of the 273,031,491 bp assembly. The distribution of sequence lengths is shown in dark grey with the plot radius scaled to the longest sequence present in the assembly (27,766,539 bp, shown in red). Orange and pale-orange arcs show the N50 and N90 sequence lengths (14,982,175 and 9,633,086 bp), respectively. The pale grey spiral shows the cumulative sequence count on a log scale with white scale lines showing successive orders of magnitude. The blue and pale-blue area around the outside of the plot shows the distribution of GC, AT and N percentages in the same bins as the inner plot. A summary of complete, fragmented, duplicated and missing BUSCO genes in the hymenoptera_odb10 set is shown in the top right. An interactive version of this figure is available at https://blobtoolkit.genomehubs.org/view/iyNomRufi1.1/dataset/CATOQH01/snail.

The final assembly has a total length of 273.0 Mb in 169 sequence scaffolds with a scaffold N50 of 15.0 Mb ( Table 1). Most (92.79%) of the assembly sequence was assigned to 16 chromosomal-level scaffolds. Chromosome-scale scaffolds confirmed by the Hi-C data are named in order of size ( Figure 2– Figure 5; Table 2). The specimen is a haploid male. The mitochondrial genome was also assembled and can be found as a contig within the multifasta file of the genome submission.

**Table 1.** Genome data for *Nomada ruficornis*, iyNomRufi1.1.

Project accession data		
Assembly identifier	iyNomRufi1.1	
Species	*Nomada ruficornis*	
Specimen	iyNomRufi1	
NCBI taxonomy ID	601849	
BioProject	PRJEB55751	
BioSample ID	SAMEA10166769	
Isolate information	iyNomRufi1, male: head and thorax (DNA sequencing) iyNomRufi2, male: whole organism (Hi-C data)	
Assembly metrics *		*Benchmark*
Consensus quality (QV)	68.9	*≥ 50*
*k*-mer completeness	100%	*≥ 95%*
BUSCO **	C:97.2%[S:96.9%,D:0.3%], F:0.5%,M:2.3%,n:5,991	*C ≥ 95%*
Percentage of assembly mapped to chromosomes	92.79%	*≥ 95%*
Sex chromosomes	-	*localised homologous pairs*
Organelles	Mitochondrial genome assembled	*complete single alleles*
Raw data accessions		
PacificBiosciences SEQUEL II	ERR10662011, ERR10168735	
Hi-C Illumina	ERR10149568	
Genome assembly		
Assembly accession	GCA_951802695.1	
Span (Mb)	273.0	
Number of contigs	227	
Contig N50 length (Mb)	7.2	
Number of scaffolds	169	
Scaffold N50 length (Mb)	15.0	
Longest scaffold (Mb)	27.8	

* Assembly metric benchmarks are adapted from column VGP-2020 of “Table 1: Proposed standards and metrics for defining genome assembly quality” from ( Rhie *et al.*, 2021). ** BUSCO scores based on the hymenoptera_odb10 BUSCO set using v5.3.2. C = complete [S = single copy, D = duplicated], F = fragmented, M = missing, n = number of orthologues in comparison. A full set of BUSCO scores is available at https://blobtoolkit.genomehubs.org/view/iyNomRufi1.1/dataset/CATOQH01/busco.

**Figure 3.**
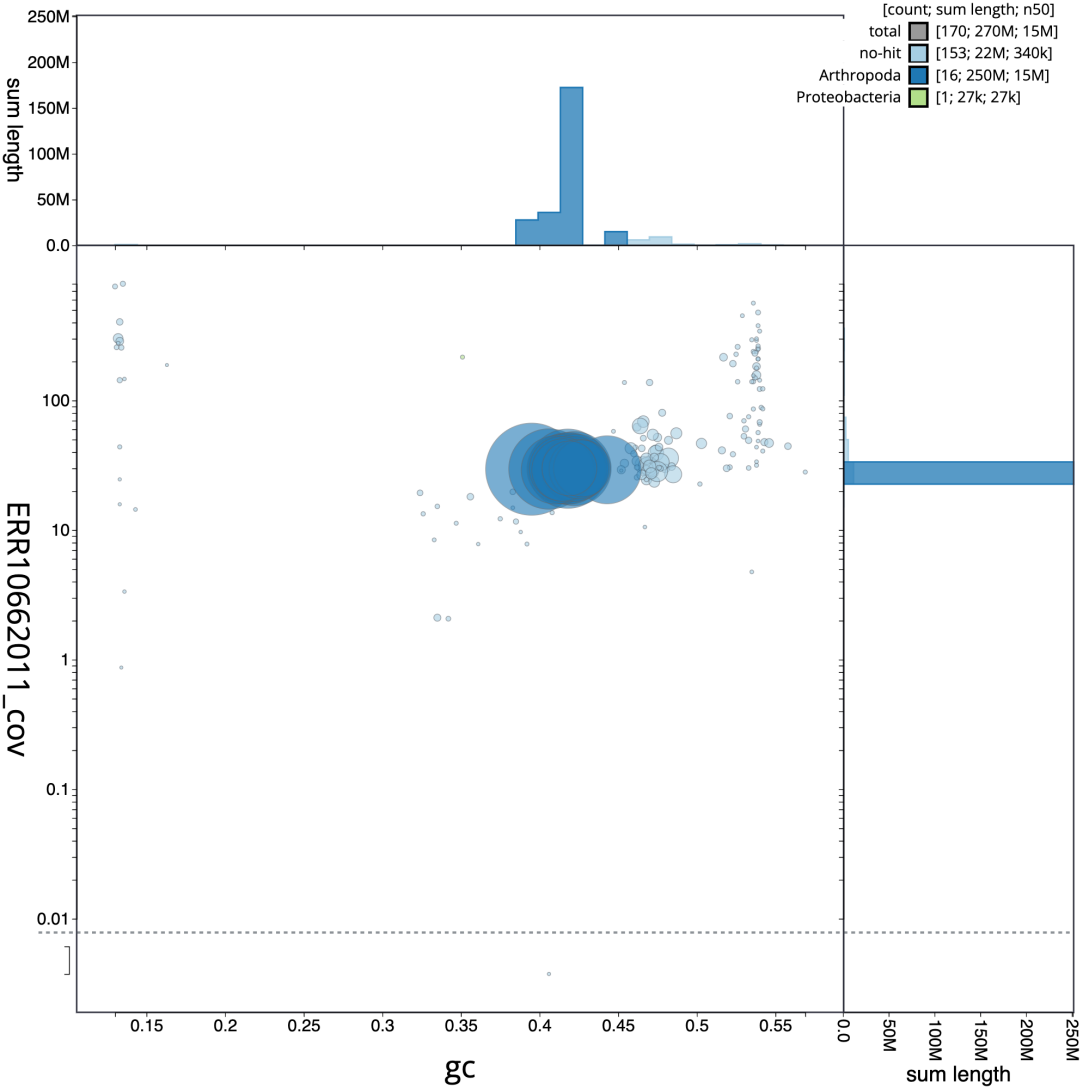
Genome assembly of *Nomada ruficornis*, iyNomRufi1.1: BlobToolKit GC-coverage plot. Scaffolds are coloured by phylum. Circles are sized in proportion to scaffold length. Histograms show the distribution of scaffold length sum along each axis. An interactive version of this figure is available at https://blobtoolkit.genomehubs.org/view/iyNomRufi1.1/dataset/CATOQH01/blob.

**Figure 4.**
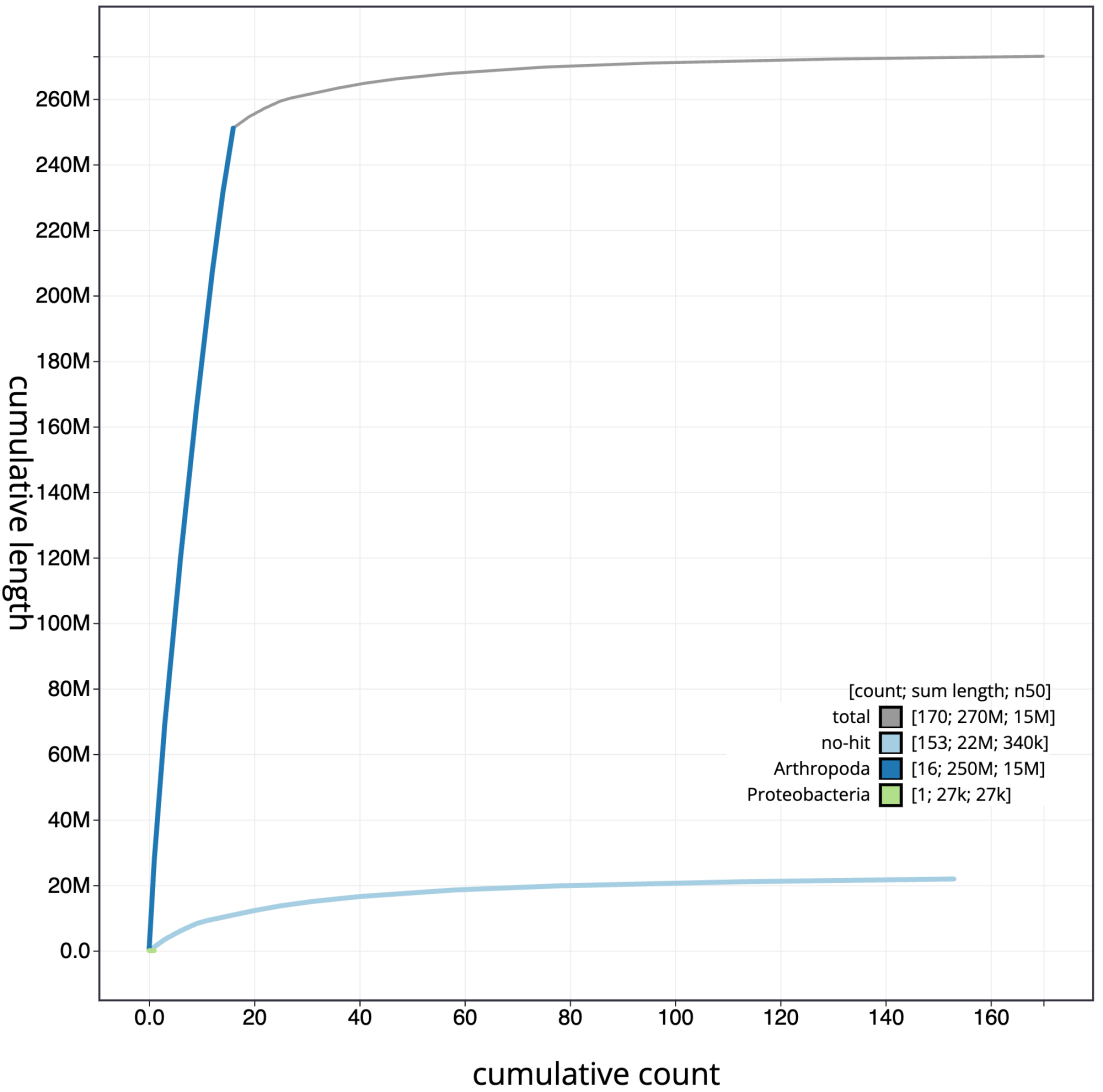
Genome assembly of *Nomada ruficornis*, iyNomRufi1.1: BlobToolKit cumulative sequence plot. The grey line shows cumulative length for all scaffolds. Coloured lines show cumulative lengths of scaffolds assigned to each phylum using the buscogenes taxrule. An interactive version of this figure is available at https://blobtoolkit.genomehubs.org/view/iyNomRufi1.1/dataset/CATOQH01/cumulative.

**Figure 5.**
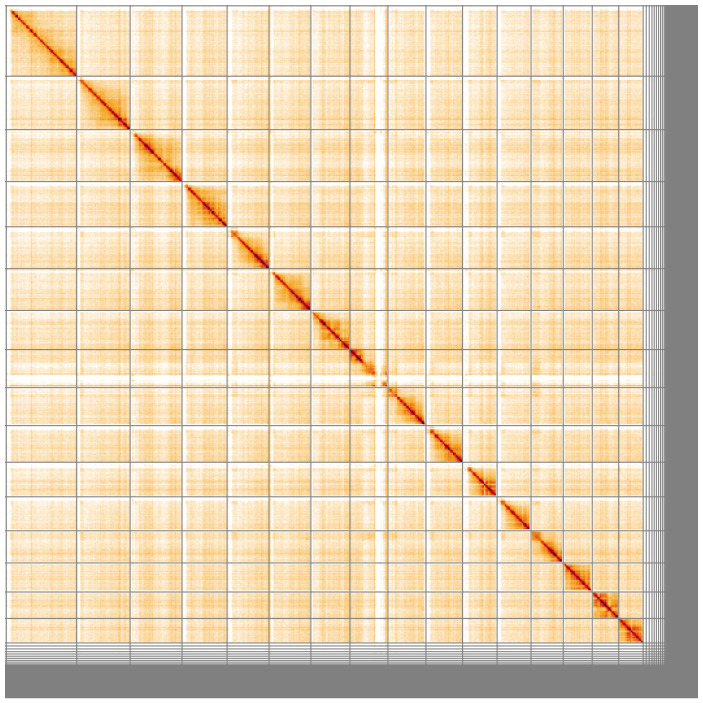
Genome assembly of *Nomada ruficornis*, iyNomRufi1.1: Hi-C contact map of the iyNomRufi1.1 assembly, visualised using HiGlass. Chromosomes are shown in order of size from left to right and top to bottom. An interactive version of this figure may be viewed at https://genome-note-higlass.tol.sanger.ac.uk/l/?d=TxoFhDkFSK6cHDFb9syBjw.

**Table 2.** Chromosomal pseudomolecules in the genome assembly of *Nomada ruficornis*, iyNomRufi1.

INSDC accession	Chromosome	Length (Mb)	GC%
OX637846.1	1	27.77	39.5
OX637847.1	2	21.06	40.5
OX637848.1	3	20.45	42.0
OX637849.1	4	17.79	42.0
OX637851.1	6	16.53	41.5
OX637852.1	7	16.5	42.0
OX637853.1	8	15.4	41.5
OX637850.1	5	14.98	44.5
OX637854.1	9	14.98	41.0
OX637855.1	10	14.45	42.5
OX637856.1	11	13.62	42.0
OX637857.1	12	13.38	41.5
OX637858.1	13	12.68	41.5
OX637859.1	14	11.44	42.5
OX637860.1	15	10.45	42.5
OX637861.1	16	9.63	42.0
OX637862.1	MT	0.02	14.5

The estimated Quality Value (QV) of the final assembly is 68.9 with *k*-mer completeness of 100%, and the assembly has a BUSCO v5.3.2 completeness of 97.2% (single = 96.9%, duplicated = 0.3%), using the hymenoptera_odb10 reference set ( *n* = 5,991).

Metadata for specimens, spectral estimates, sequencing runs, contaminants and pre-curation assembly statistics can be found at https://links.tol.sanger.ac.uk/species/601849.

## Methods

### Sample acquisition and nucleic acid extraction

Two male *Nomada ruficornis* specimens were netted in Wytham Woods, Oxfordshire (biological vice-county Berkshire), UK (latitude 51.76, longitude –1.34) on 2021-04-23. The specimens were collected and identified by Steven Falk (University of Oxford) and snap-frozen on dry ice. The specimen with ID Ox001289 (ToLID iyNomRufi1) was used for DNA sequencing, while specimen with ID Ox001295 (ToLID iyNomRufi2) was used for Hi-C scaffolding.

DNA was extracted at the Tree of Life laboratory, Wellcome Sanger Institute (WSI). The iyNomRufi1 sample was weighed and dissected on dry ice. Tissue from the head and thorax was disrupted using a Nippi Powermasher fitted with a BioMasher pestle. High molecular weight (HMW) DNA was extracted using the Qiagen MagAttract HMW DNA extraction kit. Low molecular weight DNA was removed from a 20 ng aliquot of extracted DNA using the 0.8X AMpure XP purification kit prior to 10X Chromium sequencing; a minimum of 50 ng DNA was submitted for 10X sequencing. HMW DNA was sheared into an average fragment size of 12–20 kb in a Megaruptor 3 system with speed setting 30. Sheared DNA was purified by solid-phase reversible immobilisation using AMPure PB beads with a 1.8X ratio of beads to sample to remove the shorter fragments and concentrate the DNA sample. The concentration of the sheared and purified DNA was assessed using a Nanodrop spectrophotometer and Qubit Fluorometer and Qubit dsDNA High Sensitivity Assay kit. Fragment size distribution was evaluated by running the sample on the FemtoPulse system.

### Sequencing

Pacific Biosciences HiFi circular consensus DNA sequencing libraries were constructed according to the manufacturers’ instructions. DNA sequencing was performed by the Scientific Operations core at the WSI on a Pacific Biosciences SEQUEL II (HiFi) instrument. Hi-C data were also generated from whole organism tissue of iyNomRufi2 using the Arima2 kit and sequenced on the Illumina NovaSeq 6000 instrument.

### Genome assembly, curation and evaluation

Assembly was carried out with Hifiasm ( Cheng *et al.*, 2021) and haplotypic duplication was identified and removed with purge_dups ( Guan *et al.*, 2020). The assembly was then scaffolded with Hi-C data ( Rao *et al.*, 2014) using YaHS ( Zhou *et al.*, 2023). The assembly was checked for contamination and corrected as described previously ( Howe *et al.*, 2021). Manual curation was performed using HiGlass ( Kerpedjiev *et al.*, 2018) and Pretext ( Harry, 2022). The mitochondrial genome was assembled using MitoHiFi ( Uliano-Silva *et al.*, 2023), which runs MitoFinder ( Allio *et al.*, 2020) or MITOS ( Bernt *et al.*, 2013) and uses these annotations to select the final mitochondrial contig and to ensure the general quality of the sequence.

A Hi-C map for the final assembly was produced using bwa-mem2 ( Vasimuddin *et al.*, 2019) in the Cooler file format ( Abdennur & Mirny, 2020). To assess the assembly metrics, the *k*-mer completeness and QV consensus quality values were calculated in Merqury ( Rhie *et al.*, 2020). This work was done using Nextflow ( Di Tommaso *et al.*, 2017) DSL2 pipelines “sanger-tol/readmapping” ( Surana *et al.*, 2023a) and “sanger-tol/genomenote” ( Surana *et al.*, 2023b). The genome was analysed within the BlobToolKit environment ( Challis *et al.*, 2020) and BUSCO scores ( Manni *et al.*, 2021; Simão *et al.*, 2015) were calculated.

Table 3 contains a list of relevant software tool versions and sources.

**Table 3.** Software tools: versions and sources.

Software tool	Version	Source
BlobToolKit	4.1.7	https://github.com/blobtoolkit/blobtoolkit
BUSCO	5.3.2	https://gitlab.com/ezlab/busco
Hifiasm	0.16.1-r375	https://github.com/chhylp123/hifiasm
HiGlass	1.11.6	https://github.com/higlass/higlass
Merqury	MerquryFK	https://github.com/thegenemyers/MERQURY.FK
MitoHiFi	2	https://github.com/marcelauliano/MitoHiFi
PretextView	0.2	https://github.com/wtsi-hpag/PretextView
purge_dups	1.2.3	https://github.com/dfguan/purge_dups
sanger-tol/genomenote	v1.0	https://github.com/sanger-tol/genomenote
sanger-tol/readmapping	1.1.0	https://github.com/sanger-tol/readmapping/tree/1.1.0
YaHS	yahs-1.1.91eebc2	https://github.com/c-zhou/yahs

### Wellcome Sanger Institute – Legal and Governance

The materials that have contributed to this genome note have been supplied by a Darwin Tree of Life Partner. The submission of materials by a Darwin Tree of Life Partner is subject to the **‘Darwin Tree of Life Project Sampling Code of Practice’**, which can be found in full on the Darwin Tree of Life website here. By agreeing with and signing up to the Sampling Code of Practice, the Darwin Tree of Life Partner agrees they will meet the legal and ethical requirements and standards set out within this document in respect of all samples acquired for, and supplied to, the Darwin Tree of Life Project.

Further, the Wellcome Sanger Institute employs a process whereby due diligence is carried out proportionate to the nature of the materials themselves, and the circumstances under which they have been/are to be collected and provided for use. The purpose of this is to address and mitigate any potential legal and/or ethical implications of receipt and use of the materials as part of the research project, and to ensure that in doing so we align with best practice wherever possible. The overarching areas of consideration are:

•   Ethical review of provenance and sourcing of the material

•   Legality of collection, transfer and use (national and international)

Each transfer of samples is further undertaken according to a Research Collaboration Agreement or Material Transfer Agreement entered into by the Darwin Tree of Life Partner, Genome Research Limited (operating as the Wellcome Sanger Institute), and in some circumstances other Darwin Tree of Life collaborators.

## Data Availability

European Nucleotide Archive: *Nomada ruficornis* (fork-jawed nomad bee). Accession number PRJEB55751; https://identifiers.org/ena.embl/PRJEB55751. ( Wellcome Sanger Institute, 2023) The genome sequence is released openly for reuse. The *Nomada ruficornis* genome sequencing initiative is part of the Darwin Tree of Life (DToL) project. All raw sequence data and the assembly have been deposited in INSDC databases. The genome will be annotated using available RNA-Seq data and presented through the Ensembl pipeline at the European Bioinformatics Institute. Raw data and assembly accession identifiers are reported in Table 1.

## Author information

Members of the University of Oxford and Wytham Woods Genome Acquisition Lab are listed here: https://doi.org/10.5281/zenodo.4789928.

Members of the Darwin Tree of Life Barcoding collective are listed here: https://doi.org/10.5281/zenodo.4893703.

Members of the Wellcome Sanger Institute Tree of Life programme are listed here: https://doi.org/10.5281/zenodo.4783585.

Members of Wellcome Sanger Institute Scientific Operations: DNA Pipelines collective are listed here: https://doi.org/10.5281/zenodo.4790455.

Members of the Tree of Life Core Informatics collective are listed here: https://doi.org/10.5281/zenodo.5013541.

Members of the Darwin Tree of Life Consortium are listed here: https://doi.org/10.5281/zenodo.4783558.
