# Supplementary material for: The genome sequence of the fork-jawed nomad bee, Nomada ruficornis (Linnaeus, 1758)
Source: Wellcome Open Res. 2023 Sep 6;8:394. [Version 1] doi: 10.12688/wellcomeopenres.19928.1 (PMC10697131; doi:10.12688/wellcomeopenres.19928.1)
Supplement: Peer review history

# Reviewer report: Reviewer response for version 1

**DOI:** 10.21956/wellcomeopenres.22067.r69716

**Published:** 2023-11-23

**Authors:** Trevor Sless

**Affiliations:** 1 Biology, York University, Toronto, Ontario, Canada

The submitted article reports on the complete genome of the fork-jawed nomad bee *Nomada ruficornis*, the type species of its genus. This is a significant contribution to the genomic study of cuckoo bees such as *Nomada* and also serves to further the Darwin Tree of Life Project’s broader goal. The genome assembly was generated using modern standard methods including the PacBio HiFi and Hi-C sequencing platforms, and all assembly metrics support the high quality of this new reference genome. The sequence dataset is openly accessible and will be useful for a variety of future studies.

Suggestions:

**Background:**

 “ *Nomada ruficornis* Linnaeus is a medium-sized…”

- Linnaeus author citation should be in parentheses (due to basionym *Apis ruficornis*)
- Although not strictly necessary, I feel that it is worthwhile to mention the particular taxonomic significance of *N. ruficornis* as the type species of the genus *Nomada.*

“The species can be distinguished from all other UK *Nomada species*, excluding N. fabricana…”

- “species” is unnecessarily italicized
- “ *N. fabricana*” is misspelled, it should be *N. fabriciana*

“…Palearctic to Japan (Ascher & Pickering, 2016).”

- Should probably cite newer 2020 version of Discover Life checklist rather than 2016 version as indicated here: https://www.discoverlife.org/mp/20q?act=x_guide_credit&guide=Apoidea_species

**Genome Sequence Report:**

 “Chromosome-scale scaffolds confirmed by the Hi-C data are named in order of size…”

- The chromosomes listed in Table 2 are slightly out of order, with the chromosome designated as #5 confusingly being the eighth largest. Please either rename or explain the reasoning for this.

Are sufficient details of methods and materials provided to allow replication by others?

Yes

Is the rationale for creating the dataset(s) clearly described?

Yes

Are the datasets clearly presented in a useable and accessible format?

Yes

Are the protocols appropriate and is the work technically sound?

Yes

Reviewer Expertise:

Bees, Evolution, Genomics, Phylogenetics

I confirm that I have read this submission and believe that I have an appropriate level of expertise to confirm that it is of an acceptable scientific standard.

# Reviewer report: Reviewer response for version 1

**DOI:** 10.21956/wellcomeopenres.22067.r66796

**Published:** 2023-09-21

**Authors:** Katherine A. Odanaka

**Affiliations:** 1 Department of Biology, York University, Toronto, Ontario, Canada

This manuscript reports the genome assembly of *Nomada ruficornis, *one of two bidentate *Nomada *species found in the UK. The manuscript is concise and well written and the methods are easy to follow and reproduce. The protocols listed are standard and appropriate as well and the links to the software sources, metadata, and images all function.

*Nomada fabriciana* is spelled wrong in the intro -> currently spelled *N. fabricana*

 Here are some other minor suggestions:

- One aspect that I think could be expanded on a bit more is the rationale for sequencing this bee. I agree that including it for the Darwin Tree of Life project is important but I think there could be something added to the background that could tie into pollinator diversity and/or conservation.
- Maybe add another sentence to the second paragraph of how this species differs from *N. fabriciana.*

Are sufficient details of methods and materials provided to allow replication by others?

Yes

Is the rationale for creating the dataset(s) clearly described?

Yes

Are the datasets clearly presented in a useable and accessible format?

Yes

Are the protocols appropriate and is the work technically sound?

Yes

Reviewer Expertise:

Nomada evolution

I confirm that I have read this submission and believe that I have an appropriate level of expertise to confirm that it is of an acceptable scientific standard.
